# Catastrophic health expenditure due to hospitalisation for COVID-19 treatment in India: findings from a primary survey

**DOI:** 10.1186/s13104-022-05977-6

**Published:** 2022-03-03

**Authors:** Samir Garg, Kirtti Kumar Bebarta, Narayan Tripathi, C. Krishnendhu

**Affiliations:** State Health Resource Centre, Raipur, Chhattisgarh India

**Keywords:** Hospitalisation, Expenditure, Out of pocket, Catastrophic expenditure, Financial protection, COVID-19, Private sector, India, Insurance

## Abstract

**Objective:**

The COVID-19 pandemic has caused widespread illness and a significant proportion of the infected required hospitalisation for treatment. People in developing countries like India were vulnerable to high hospitalisation costs. Despite its crucial importance, few primary studies are available on this aspect of the pandemic. This study was aimed at finding out the out of pocket expenditure (OOPE) and incidence of catastrophic expenditure on hospitalisation of persons infected with COVID-19. A primary survey of 492 randomly selected hospitalisations of individuals tested positive for COVID-19 in high-burden districts during August to November 2020 was carried out telephonically in Chhattisgarh state of India.

**Results:**

Public hospitals accounted for 69% of the hospitalisations for COVID-19 treatment. Mean OOPE per hospitalisation was Indian Rupees (INR) 4871 in public hospitals and INR 169,504 in private hospitals. Around 3% of hospitalisations in public hospitals and 59% in private hospitals resulted in catastrophic expenditure, at a threshold of 40% of non-food annual household expenditure. Enrolment under publicly or privately funded health insurance was not effective in curtailing OOPE. Multivariate analysis showed that utilisation of private hospitals was a key determinant of incurring catastrophic expenditure.

**Supplementary Information:**

The online version contains supplementary material available at 10.1186/s13104-022-05977-6.

## Introduction

The COVID-19 pandemic has affected countries across the world and has caused widespread illness, mortality and economic catastrophe [1–3]. Globally, a large number of individuals infected with COVID-19 had to be admitted in hospitals for treatment [4]. Researchers have expressed a concern that a large number of households in developing countries including India were vulnerable to catastrophic expenditure for COVID-19 related hospitalisations due to the existing dependence of health systems on Out of Pocket Expenditure (OOPE) [5–8]. India was among the worst affected countries in the world in terms of the number of COVID-19 infected persons [4]. The health system in India is a mixed one with a substantial presence of for-profit private sector alongside public facilities [9]. OOPE constitutes around 60% of the Total Health Expenditure in Indian health system [10]. In such a situation, it is of crucial importance to know the financial burden borne by the households on hospitalisation for COVID-19 treatment.

In terms of policy responses in India, the central and state governments tried to expand the capacity of public hospitals and offered free services for COVID-19 treatment [11]. India has a Publicly Funded Health Insurance (PFHI) programme known as Ayushman Bharat—Pradhan Mantri Jan Arogaya Yojana (PMJAY) to cover in-patient care costs in public as well as private hospitals [11, 12]. In addition, governments in many states of India declared price ceilings for COVID-19 related care in private hospitals [13–19]. However, not much is known regarding the effectiveness of above measures.

The current study was aimed at finding out the OOPE and incidence of catastrophic health expenditure (CHE) for hospitalisations of COVID-19 cases.

## Main text

### Materials and methods

#### Study setting

The study was conducted in the Indian state of Chhattisgarh. It is one of the poorest states in India and has a population of around 29 million [20]. From August 2020 onwards, government allowed private hospitals in the state to admit COVID-19 cases [21]. Home Isolation was allowed for COVID-19 positive cases with mild or no symptoms [22]. The state had universal enrolment under its PFHI schemes that included PMJAY [23, 24]. Starting from April 2020, PMJAY had allowed the states to include testing and hospitalisation for COVID-19 under its cover [12]. Following the above decision, the state invited private hospitals to get empanelled to provide hospitalisation care for COVID-19 cases at the following prices per day: Indian Rupees (INR) 2200 for care in general ward, INR 3750 for care in Intensive Care Unit (ICU) and INR 6750 for ICU with ventilation support [25]. However, none of the private hospitals came forward to get empanelled for COVID-19 care under the above scheme [23].

In August 2020, the state government declared a ceiling on prices that the private hospitals could charge from COVID-19 patients. The per-day ceiling was INR 6200 for care at general ward with oxygen support, INR 12,000 for care at ICU, INR 17,000 for ventilator support [13].

Study design and sampling: The study was based on a cross-sectional survey of COVID-19 cases. The survey was done in December, 2020 by the State Health Resource Centre, a technical agency working for the state’s department of health. A structured quantitative questionnaire was used. Due to poor feasibility of conducting in-person interviews during the pandemic, the survey was carried out telephonically. The survey and its quality-assurance were informed by the guidance available for conducting phone-based surveys in developing countries during the COVID-19 pandemic [26–29]. In case of participants below 18 years age, either of the parents was interviewed.

The sample consisted of COVID-19 positive individuals who got hospitalised for its treatment. COVID-19 positive was taken as those confirmed for COVID-19 using diagnostics approved by Indian government. Government rules made it mandatory for all providers to record all tests done for COVID-19 on a centralized online database. A list of 181,616 confirmed cases of COVID-19 in Chhattisgarh during the study period (August to November 2020) was obtained from the government. The top three districts in the state in terms of most number of cases were selected for survey. The minimum sample size required at 95% confidence was calculated as 385 hospitalisations. Expecting one-third of the COVID-19 cases to be hospitalised, a sample of three times i.e. 1155 cases was needed. In order to allow for a refusal rate of 50% in the telephonic survey, 2310 cases were taken using systematic random selection. The survey was able to complete interviews of 1294 individuals out of the above list. The above interviews yielded 492 participants who had been hospitalised for COVID-19 treatment and they formed the sample for this study.

OOPE for hospitalisation was taken as all medical expenses paid in cash by the patient/family including all charges paid to hospital and any medicines bought. The expenditure on transportation for hospitalisation was asked separately. The expenditure on testing for COVID-19 before hospitalisation was also asked separately. There were no cash-reimbursements received by the patients.

Financial Protection was measured in terms of catastrophic health expenditure (CHE) as proposed by Wagstaff and Doorslaer [30]. The survey collected data on monthly consumption expenditure on food and non-food purposes. The incidence of CHE was calculated using a threshold based on a proportion of annual non-food consumption expenditure. A threshold of 40% of concerned household’s annual non-food consumption expenditure was taken and named CHE40.This is a commonly used measure of CHE [30].

The list of study variables is given in Additional file 1: Table S1.

Confidence Intervals (CI) were computed for mean at 95% and reported in parentheses. Multi-variate logistic regression was used to find out determinants of CHE40. As recommended in existing studies, quantile regression was applied for OOPE as it offers advantages in addressing any skew or extreme values in the OOPE data [31]. For comparison, linear regression was also applied for OOPE. All analyses were carried out using STATA 15.

#### Ethical considerations

The ethics approval for the study was obtained from the Institutional Ethics Committee of the State Health Resource Centre, Chhattisgarh [Reference No. SHRC-04-2020]. The consent form was read out to each participant. The interview was started only when an explicit consent was provided by participants. A log was maintained for every call—name of caller, date and time, note that the prescribed text was used for asking consent, record of participant’s response and signature of caller. In order to ensure confidentiality and quality, an in-house call-centre was used. The calls were supervised closely to ensure adherence to the protocol. The log of each caller was examined and signed daily by the concerned supervisor. The dataset was completely anonymised.

### Results

Of the COVID-19 positive individuals interviewed, 83.1% (80.9–85.1%) had been tested for COVID-19 by government providers. Among the COVID-19 positive individuals interviewed, 38% (35.440.7%) had got hospitalised.

The socio-demographic characteristics of the hospitalised COVID-19 positive individuals are given in Table 1.

**Table 1:** Socio-demographic characteristics of individuals interviewed and individuals hospitalised among them

Characteristics and categories	Hospitalised individuals N = 492(%)
Place of residence	
Rural	134 (27.24)
Urban	358 (72.76)
Age (years)	
0–14	15 (3.08)
15–39	231 (47.43)
40–59	163 (33.47)
Above 60	78 (16.02)
Sex	
Male	342 (69.51)
Female	150 (30.49)
Education	
Not literate	24 (4.79)
Primary	62 (12.50)
High school	87 (17.71)
12th standard	99 (20.21)
Graduation and above	220 (44.79)
Per-capita household expenditure quintile	
Quintile 1 (poorest)	119 (25.93)
Quintile 2	73 (15.90)
Quintile 3	95 (20.70)
Quintile 4	81 (17.65)
Quintile 5	91 (19.83)
Household size category	
Up to 5 members	304 (61.79)
Above 5 members	188 (38.21)
Private insurance	
Enrolled	57 (11.59)

The mean annual household expenditure of the hospitalised participants was INR 339,643 (273,607–405,679) and its median value was INR 267,000.

Around 12% of the hospitalised individuals were enrolled under private health insurance. Private health insurance in Indian context refers to voluntary insurance bought by individuals from private insurers to cover costs of utilizing private hospitals [32].

Among those who got hospitalised, 69.3% (65.1–73.4%) were hospitalised in public facilities and the rest utilised for-profit private hospitals. Among hospitalisations, 15.6% were shorter than a week, 68.8% were 7 to 13 days long and 15.5% were 2 weeks or longer. The mean duration of hospitalisation was around 10 days for both public and private hospitals. Of the hospitalised, 7.5% utilised ventilator support, 10.8% used oxygen (without ventilator) and 16.5% received anti-viral injections.

The mean expenditure incurred by patients for testing for COVID-19 was INR 130 (62–197) for public hospitals and INR 2003 (1359–2647) for private hospitals. The mean expenditure incurred by patients for transportation for hospitalisation was INR 280 (135–424) for public hospitals and INR 791 (360–1222) for private hospitals.

The mean OOPE on hospitalisation was INR 169,504 (142,094–196,914) in private hospitals and INR 4871 (3068–6674) in public hospitals. Among the hospitalisations in private hospitals, 87.1% (80.2–94.1%) involved charges exceeding the price caps declared by government.

The quantile regression for OOPE showed that utilising private hospitals involved significantly greater OOPE than public hospitals (Table 2). Receiving ventilator support or anti-viral injections involved greater OOPE. Enrolment under the private health insurance did not have a significant association with OOPE. The linear regression model showed a similar pattern (Additional file 2: Table S2).

**Table 2:** Quantile regression for size of OOPE on COVID-19 hospitalisation

	OOPE	Coef	SE	P value	95% CI	
Residence	Rural	1				
	Urban	0	6327	1	− 12,541	12,541
Age	0–14 years	1				
	15–39 years	0	16,819	1	− 33,320	33,320
	40–59 years	0	16,607	1	− 32,820	32,820
	Above 60	0	17,330	1	− 34,207	34,207
Sex	Male	1				
	Female	0	5906	1	− 11,612	11,612
Education	Not literate	1				
	Primary	0	9617	1	− 18,907	18,907
	High school	0	10,129	1	− 19,914	19,914
	12th standard	0	10,489	1	− 20,621	20,621
	Graduation and above	0	14,725	1	− 28,950	28,950
Household size	Up to 5 members	1				
	Above 5 members	0	5925	1	− 11,648	11,648
Per capita household expenditure quintile	Poorest	1				
	Poor	0	8744	1	− 17,191	17,191
	Middle	0	8412	1	− 16,538	16,538
	Rich	0	8974	1	− 17,644	17,644
	Richest	1000	9320	0.91	− 17,323	19,323
Type of hospital	Public	1				
	Private	133,000	6720	< 0.01	119,787	146,213
Duration of hospitalisation		0	613	1.1	− 1206	1206
Ventilator	Used	19,000	12,060	0.11	− 4710	42,710
Anti-viral injection	Used	40,000	8767	< 0.01	22,763	57,237
Oxygen (without ventilator)	Used	5000	9347	0.5	− 13,376	23,376
Private insurance	Insured	− 2000	8888	0.8	− 19,473	15,473

No. of observations: 413; pseudo R2: 0.37

Among those hospitalised in public hospitals, 3.2% (1.8–5.7%) incurred CHE40. Among those hospitalised in private hospitals, 58.9% (50.5–66.74%) incurred CHE40. Overall, 20.3% (16.9–24.1%) of the hospitalised incurred CHE40. Using private hospitals involved significantly greater likelihood of catastrophic expenditure than those using public hospitals (Table 3). Enrolment in private health insurance did not have a significant association with CHE40.

**Table 3:** Logistic regression for CHE40 on COVID-19 hospitalisation

	Odds ratio	SE	p value	95% CI	
Residence					
Rural	1				
Urban	1.48	0.76	0.44	0.54	4.07
Age (years)					
0–14	1				
15–39	1.13	2.01	0.95	0.03	37.20
40–59	0.90	1.58	0.95	0.03	28.36
Above 60	1.30	2.31	0.88	0.04	42.06
Sex					
Male	1				
Female	3.09	1.36	0.01	1.31	7.30
Education					
Not literate	1				
Primary	0.79	0.53	0.72	0.21	2.93
High school	0.65	0.47	0.55	0.16	2.70
12th standard	0.83	0.62	0.81	0.19	3.58
Graduation and above	0.21	0.34	0.33	0.01	4.96
Household size					
Up to 5 members	1				
Above 5 members	0.44	0.18	0.05	0.19	1.00
Per capita household expenditure quintile					
Poorest	1				
Poor	0.54	0.39	0.39	0.13	2.20
Middle	0.60	0.40	0.45	0.16	2.22
Rich	0.95	0.61	0.93	0.27	3.36
Richest	0.36	0.25	0.14	0.10	1.38
Type of hospital					
Public	1				
Private	59.77	30.47	0.01	22.00	162.34
Duration of hospitalisation	1.06	0.04	0.14	0.98	1.15
Ventilator					
Used	3.74	2.52	0.04	1.00	14.01
Anti-viral injection					
Used	2.34	1.05	0.06	0.97	5.64
Oxygen (without ventilator)					
Used	2.26	1.09	0.09	0.88	5.82
Private insurance					
Insured	0.67	0.31	0.39	0.27	1.68

No. of observations: 413; pseudo R2: 0.5

### Discussion

The study period of August to November 2020 corresponded to the first wave of surge in COVID-19 infections in India [33]. Those admitted in private hospitals incurred thirty-five times larger OOPE than the admissions in public hospitals. The adjusted model showed that hospitalisation in private facilities was far more likely to cause catastrophic expenditure than the public facilities. This confirms the apprehensions regarding the possibility of catastrophic expenditure due to COVID-19, especially for hospitalisations in the private sector [6, 34, 35].

The current study found that private insurance was ineffective in reducing the OOPE significantly. A recent study has examined the claims data of private insurance in India and expressed similar doubts regarding its effectiveness [36]. PFHI schemes in Indian context, including the national flagship programme PMJAY, have been found to be largely ineffective in reducing OOPE or CHE for inpatient care [20, 37–39]. The current study found that the PMJAY scheme could not cover COVID-19 care in private hospitals in Chhattisgarh in 2020.

In addition to existing laws on regulation of private hospitals, government enjoyed further powers under the epidemic related law invoked for COVID-19 [3]. The price ceilings announced by Chhattisgarh state were around three times greater than the prices it offered under PFHI. Yet, the private hospitals continued to charge above the price caps. According to grey literature, the price controls introduced by other states in India for COVID-19 hospitalisations were also quite ineffective [35]. This reflects the long-standing difficulties in achieving price regulation in private hospitals in India [32, 40–43].

A study from Peru has reported high OOPE for COVID-19 hospitalisations and ineffectiveness of health insurance [44]. Studies from USA have found significant OOPE for COVID-19 hospitalisations among the insured [45, 46]. Examples of price gouging by private hospitals for COVID-19 care have been found in many countries in Africa [47]. In many health systems, the providers are known to wield a lot more power compared to the patients [41, 48]. This power asymmetry seems to have worsened during the pandemic when widespread panic prevailed.

Some have advocated that governments should purchase care for COVID-19 from private sector hospitals [49–53]. The current study suggests that such strategies are unlikely to succeed in Indian context. Publicly provided care was found to be effective in ensuring financial protection. However, as public hospitals diverted a significant amount of their capacity to COVID-19 care, the availability of care for other ailments might have suffered [54].

For curtailing OOPE, the current study suggests the need for better regulation of charging in private hospitals. There is a need to strengthen capacity of public sector to manage a surge of hospitalisations during emergencies while maintaining other essential services.

## Limitations

The usual limitations of cross-sectional studies apply. Severity of the illness could not be assessed though the use of relevant medical procedures was taken into account. Quality of healthcare is an important dimension but it could not be addressed. There is a possibility that the sample included a few cases of more than one hospitalisation from the same family.

## Supplementary Information

**Additional file 1: Table S1.** List of study variables. List of variables used in the study.

**Additional file 2: Table S2.** Linear regression for determinants of OOPE on COVID-19 hospitalisation. Results of linear regression for determinants of Out of Pocket Expenditure (OOPE) on COVID-19 hospitalisation.

## Data Availability

The datasets used and/or analysed during the current study available from the corresponding author on reasonable request.

## Abbreviations

CHE: Catastrophic health expenditure
CHE40: Catastrophic health expenditure at threshold of 40% of non-food expenditure
INR: Indian Rupees
OOPE: Out-of-pocket expenditure
PFHI: Publicly Funded Health Insurance
PMJAY: Pradhan Mantri Jan Arogaya Yojana
USA: United States of America
