# Supplementary material for: Catastrophic health expenditure due to hospitalisation for COVID-19 treatment in India: findings from a primary survey
Source: BMC Res Notes. 2022 Mar 3;15:86. doi: 10.1186/s13104-022-05977-6 (PMC8892404; doi:10.1186/s13104-022-05977-6)
Supplement: Supplementary file 1 — Additional file 1: Table S1. List of study variables. List of variables used in the study. [file 13104_2022_5977_MOESM1_ESM.docx]

| **Additional file 1: Table S1. List of Study Variables** | |  |
| --- | --- | --- |
|  |  |  |
|  |  |  |
| **Variable** | **Description** | **Category** |
| Place of residence | Place of Residence of Individual | Rural |
|  |  | Urban |
| Age Category | Age Category of Individual | 0-14 Years |
|  |  | 15-39 Years |
|  |  | 40-59 Years |
|  |  | Above 60 |
| Sex | Sex of Individual | Male |
|  |  | Female |
| Education | Education Category of Individual | Not Literate |
|  |  | Primary |
|  |  | Higher secondary |
|  |  | 12th |
|  |  | Graduation and above |
| Household Size category | Size of Individual's Household | Upto 5 members |
|  |  | Above 5 members |
| Per Capita Household Expenditure Quintile | Quintiles of Per-capita Consumption Expenditure of Individual's Household | Poorest |
|  |  | Poor |
|  |  | Middle |
|  |  | Rich |
|  |  | Richest |
| Private Insurance | Whether the individual had private insurance for in-patient care | Yes/No |
| Type of Hospital | Type of Hospital in which hospitalisation took place | Public Hospital |
|  |  | Private Hospital |
| Duration of Hospitalisation | Duration of Hospitalisation Episode in no. of days | Continuous |
| Oxygen use (without ventilator) | Whether the Individual utilised oxygen support (without the use of ventilator) during hospitalisation for Covid-19 | Availed |
|  |  | Not Availed |
| Ventilator Use | Whether the Individual utilised ventilator support during hospitalisation for Covid-19 | Availed |
|  |  | Not Availed |
| Anti-viral Injection use | Whether the Individual received anti-viral injection/s during hospitalisation for Covid-19 | Availed |
|  |  | Not Availed |
| OOPE | Out of Pocket Expenditure (OOPE) in hospital in Indian Rupees (INR) | Continuous |
| CHE40 | Catastrophic Health Expenditure at 40% threshold of Non-food consumption expenditure (OOPE > 40% of Annual Non-food Consumption Expenditure of concerned Household) | Yes |
|  |  | No |
| Expenditure on Transport | Expenditure on Transport for hospitalisation in INR | Continuous |
| Expenditure on Testing | Expenditure on Testing for COVID-19 in INR | Continuous |
