# Supplementary material for: Catastrophic health expenditure due to hospitalisation for COVID-19 treatment in India: findings from a primary survey
Source: BMC Res Notes. 2022 Mar 3;15:86. doi: 10.1186/s13104-022-05977-6 (PMC8892404; doi:10.1186/s13104-022-05977-6)
Supplement: Supplementary file 2 — Additional file 2: Table S2. Linear regression for determinants of OOPE on COVID-19 hospitalisation. Results of linear regression for determinants of Out of Pocket Expenditure (OOPE) on COVID-19 hospitalisation. [file 13104_2022_5977_MOESM2_ESM.docx]

**Additional file 2: Table S2. Linear Regression for determinants of OOPE on Covid-19 hospitalization**

No. of Observations: 413 R-squared: 0.59

|  | **OOPE** | **Coef.** | **SE** | **P Value** | **95% CI** | |
| --- | --- | --- | --- | --- | --- | --- |
| Residence | Rural | 1 |  | | | |
|  | Urban | 3517 | 9285 | 0.71 | -14738 | 21772 |
| Age | 0-14 Years | 1 |  | | | |
|  | 15-39 Years | -16647 | 24670 | 0.50 | -65148 | 31855 |
|  | 40-59 Years | -15327 | 24300 | 0.53 | -63101 | 32446 |
|  | Above 60 | 14484 | 25326 | 0.57 | -35308 | 64277 |
| Sex | Male | 1 |  | | | |
|  | Female | 10065 | 8598 | 0.24 | -6838 | 26968 |
| Education | Not Literate | 1 |  | | | |
|  | Primary | 5288 | 13998 | 0.71 | -22233 | 32810 |
|  | High School | 4285 | 14744 | 0.77 | -24702 | 33272 |
|  | 12th Standard | -773 | 15268 | 0.96 | -30790 | 29244 |
|  | Graduation and above | -36332 | 21434 | 0.09 | -78473 | 5808 |
| Household Size | Upto 5 members | 1 |  | | | |
|  | Above 5 members | 3016 | 8624 | 0.73 | -13939 | 19971 |
| Per Capita Household Expenditure Quintile | Poorest | 1 |  | | | |
|  | Poor | -1805 | 12728 | 0.89 | -26828 | 23218 |
|  | Middle | -13947 | 12244 | 0.26 | -38019 | 10126 |
|  | Rich | 1679 | 13063 | 0.90 | -24005 | 27362 |
|  | Richest | 317 | 13566 | 0.98 | -26355 | 26989 |
| Type of Hospital | Public | 1 |  | | | |
|  | Private | 128479 | 9782 | 0.01 | 109246 | 147712 |
| Duration of Hospitalisation |  | 2567 | 893 | 0.01 | 811 | 4324 |
| Ventilator | Used | 126917 | 17554 | 0.01 | 92405 | 161429 |
| Anti-viral Injection | Used | 47447 | 12762 | 0.01 | 22356 | 72538 |
| Oxygen (without ventilator) | Used | 3098 | 13605 | 0.82 | -23651 | 29846 |
| Private insurance | Insured | -18127 | 12937 | 0.16 | -43561 | 7308 |
